# A Wearable and Wireless Gas-Sensing System Using Flexible Polymer/Multi-Walled Carbon Nanotube Composite Films

**DOI:** 10.3390/polym9090457

**Published:** 2017-09-18

**Authors:** Jin-Chern Chiou, Chin-Cheng Wu

**Affiliations:** 1 Department of Electrical Engineering, National Chiao Tung University, 1001 University Road, Hsinchu City 30010, Taiwan; chiou@mail.nctu.edu.tw; 2 Institute of Electrical and Control Engineering, National Chiao Tung University, 1001 University Road, Hsinchu City 30010, Taiwan

**Keywords:** polymer/multi-walled carbon nanotube composites, wearable device, wireless device, air pollution

## Abstract

In this study, an integrated flexible gas sensor was developed based on a polymer/multi-walled carbon nanotube composite film by using Bluetooth wireless communication/interface technology. Polymer/multi-walled carbon nanotube composite films were deposited over a polyimide flexible substrate for building a gas sensor array by using a drop-casting method. Sensor response was acquired through interdigitated electrodes and multi-channel sensor boards, which were linked to a Bluetooth wireless transceiver. Additionally, a double-spiral-shaped heater was built into the backside of the gas sensor array as a thermostat to protect it from the influence of ambient temperature. Multi-channel sensing responses were read on a display screen via a smartphone application (app). The advantages of this system include light weight, low cost, highly integrated sensors, wireless telecommunication, and real-time functioning. Thus, it is a promising candidate for deployment in a wearable gas-sensing system used to study air pollution.

## 1. Introduction

Air pollution is a global issue that seriously impacts humans and the environment. According to the World Health Organization (WHO) 2016 air quality model reports, more than 90% of the world’s population breathes air that is polluted beyond the limits specified by WHO [1]. The most common pollutants in air are carbon monoxide (CO), sulfur dioxide (SO_2_), ozone (O_3_), particulate matter (PM), and volatile organic compounds (VOCs). Specifically, CO is generated when carbon-containing materials are burnt. A major source of CO is the combustion of fossil fuels in vehicles [2]. Additionally, SO_2_ is produced mainly by the oxidation of sulfur-containing materials. Combustion of fossil fuels in power plants and refinery facilities is a major source of SO_2_. Bad O_3_ is associated with chemical reactions between oxides of nitrogen (NO_x_) and VOCs. It is produced when pollutants are emitted by cars, power plants, industrial boilers, and refinery plants in the presence of sunlight [1]. Man-made sources of PM2.5 and PM0.1 particles dominate the total concentration of pollutants. Emissions of PM2.5 and PM0.1 particles can be ascribed mainly to vehicular exhaust, road dust, and forest fires [3,4]. Principal sources of VOCs include paints, paint strippers and other solvents, wood preservatives, aerosol sprays, cleansers and disinfectants, moth repellents and air fresheners, and building materials and furnishings [5].

Furthermore, VOCs are typically defined as compounds with an initial boiling point that is less than or equal to 250 °C at the standard atmospheric pressure of 101.3 kPa. Various toxic VOCs exert a toll on the environment and cause respiratory diseases. Based on their chemical structures, VOCs are categorized into several types, including alkanes, aromatic hydrocarbons, olefins, halogenated hydrocarbons, esters, aldehydes, and ketones. Individuals facing long-term exposure to 100 ppb formaldehyde could develop nasal cancer [6]. Benzene may also cause acute myeloid leukemia (acute non-lymphocytic leukemia) [7]. A few studies provide strong evidence that toluene affects the central nervous system (CNS) [8]. To evaluate the concentration of toxic VOCs, several precision methods and instruments have been designed and examined. They include gas chromatography (GC) [9], high-pressure liquid chromatography (LC) [10], the gas chromatography–mass spectrometry (GC–MS) coupled method [11], ion mobility spectroscopy [12], atomic emission detection (AED) [13], and Fourier transform infrared (FTIR) spectroscopy [14]. Most of these methods provide high sensitivity and high reliability. Their major disadvantages include high costs, time-consuming processes, and requirement for advanced techniques.

Wearable devices with integrated wireless technology have the potential to integrate an end-user with the Internet of Things, and offer healthcare and long-term, real-time information for personal measurement [15]. The features of wearable devices include light weight, low cost, highly integrated sensors, and wireless telecommunication. Several different types of miniaturized gas sensors have been investigated, such as electrochemical sensors [16], electrochemical sensors of surface acoustic wave (SAW) devices [17,18], quartz crystal microbalances (QCM) [19,20], and chemiresistive gas sensors [21,22,23,24,25,26]. Polymer/MWCNT composite sensors correspond to chemiresistive-type gas sensors owing to their unique electrical, physical, and chemical properties that facilitate the development of sensitive devices for use in wearable gas-sensing applications. Gas sensors using conducting polymers as the sensing layer show excellent response in wearable device applications at room temperature [27,28,29]. However, the range of ambient temperature varies greatly. For instance, the difference in ambient temperature between summer and winter is 20–30 °C, and the climate patterns of the northern and the southern hemispheres are opposite. The influence of ambient temperature restricts the application of a wearable polymer-based gas sensor system. The effects of temperature on polymer/MWCNT composite gas sensors have been investigated in our previous study [30,31]. To solve this problem, thermal treatment is a straightforward method used to decrease the influence of ambient temperature on polymer/MWCNT composite gas sensors. In previous studies, three different polymers, namely, polyethylene oxide (PEO), ethylcellulose (EC) and polyvinylpyrrolidone (PVP), were selected to manufacture a flexible polymer/MWCNT composite sensing films for gas sensor array that was exposed to 1.5% ethanol at different operating temperatures. The response of each polymer/MWCNT composite film indicated that higher operating temperature could mitigate the influence of ambient temperature but reduce the response. Review of the data from the previous experiments led to a program to improve the flexible polymer/MWCNT composite gas sensor array for the possibility of wearable device application. Improvement considerations include the selection of polymers for high gas selectivity, the suitable operating temperature to immune ambient temperature influence, and methods of data transmission.

In this study, poly (α-methylstyrene) (PMS) was conducted primarily on increasing gas selectivity in the sensor array. The suitable operating temperature was considered with power consumption and heating–cooling profile. This study also focused on the development of a stand-alone wearable and wireless gas-sensing system based on a Bluetooth module. The polymer/MWCNT composite gas sensor was accompanied the smartphone applications were programmed in the Android environment. To verify the feasibility of recognizing the selectivity of different gases, the sensor array was exposed to various gases, including ammonia, nitrogen dioxide, and toluene vapors. The resulting sensor array response patterns show that the system has good selectivity to the target gas. The real-time sensing and to display the sensor response were demonstrated on a smartphone.

## 2. Materials and Methods

**Figure 1:**
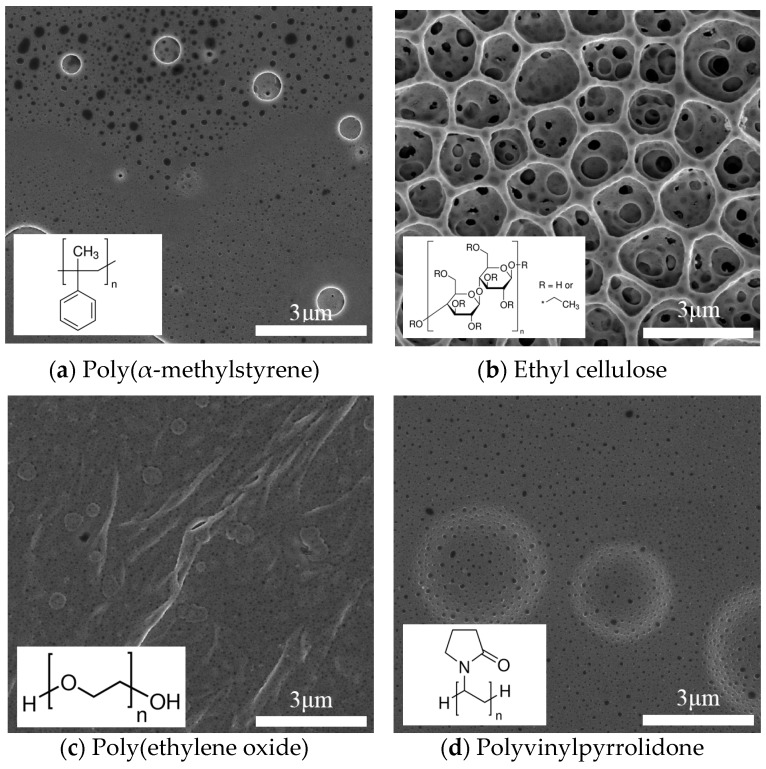
Schematic of chemical structural formulae of four polymers used herein: (**a**) PMS; (**b**) PEO; (**c**) EC; and (**d**) PVP.

The sensing film was prepared drop-casting to form the bilayer sensor structure of the gas sensor array. Four different polymers were selected, namely, ethylcellulose (200697, SIGMA-ALDRICH, St. Louis, MO, USA), polyethylene oxide (43678, Alfa Aesar, Haverhill, MA, USA), poly(α-methylstyrene) (81516, SIGMA-ALDRICH, St. Louis, MO, USA), and polyvinylpyrrolidone (PVP 10, SIGMA-ALDRICH, St. Louis, MO, USA). Figure 1a–d show the morphology of a selected polymer that was examined using a scanning electron microscope (SEM) (NOVA NANO SEM 450, FEI Co., Hillsboro, OR, USA) operated at an accelerating voltage of 10 kV.

**Figure 2:**
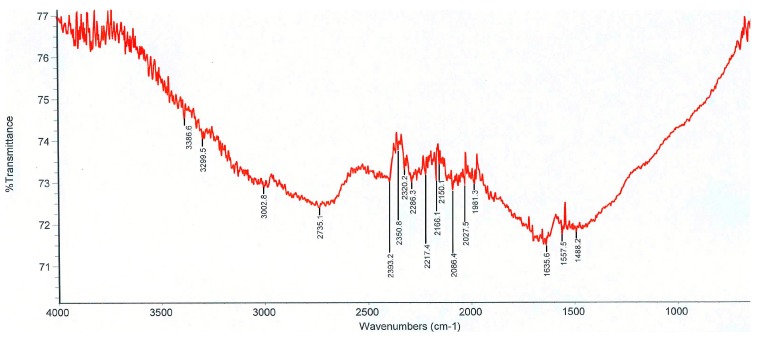
FTIR spectra of multi-walled carbon nanotube.

The MWCNTs used in this study have outer diameters ranging from 2 to 6 nm and lengths of 10 to 12 μm, and they were purchased from XinNano Materials, Inc, Taoyuan, Taiwan. Figure 2 shows the FTIR spectra of the MWCNTs. The nature of the chemical bonds formed was recorded via FTIR spectra (PERKIN ELMER Spectrum GX, Waltham, MA, USA) in the range of 4000–400 cm^−1^ for investigation purposes. Figure 2 shows the characteristic peaks of MWNT at 1488.2 cm^−1^ (–COOH), 1635.6 cm^−1^ (C=C), and 2735.1 cm^−1^ (bonded OH in carboxylic acid).

The drop-casting method was implemented to fabricate the polymer/MWCNT composite films. The fabrication process has been described in a preliminary study [31], and it is presented briefly here as follows:

(1) Approximately 2 μL of MWCNT (60 ppm) is first dropped on the interdigitated electrode by using a microjet.
(2) The solvent is evaporated, and the MWCNT film is furnished for 6 h at 70 °C.
(3) Approximately 2 μL solution of the selected polymers is dropped on the MWCNT layer by using a microjet.
(4) The solvent is evaporated completely, and the selected polymer film is furnished for 24 h at 60 °C.
(5) After performing the aforementioned casting steps, the resistance of each sensor is confirmed to limit the value within 1–50 kΩ.

## 3. Gas-Sensing System Design

### 3.1. Flexible Gas Sensor Array

The flexible polymer/MWCNT composite gas sensor was based on the flexible printed circuit (FPC) industry technology offers several advantages, including cost effectiveness, light weight, and flexibility, which is essential given its potential for integration in wearable consumer products. The gas sensor array comprised four different types of polymer/MWCNT composite sensing films and a platinum resistance temperature detector (RTD) arranged in a 3 × 3 matrix pattern. When the polymer concentration was higher than 1 wt %, the polymer was slightly sticky and would not easily form a uniform sensing film via drop-casting. When the polymer concentration was lower than 1 wt %, it was difficult to form a complete polymer film above the MWCNT surface. Therefore, the selected polymers were used at a concentration of 1 wt %. The sensor name and the relative element numbers are presented in Table 1. Each type of sensor was arranged in one of the rows in the matrix.

**Figure 3:**
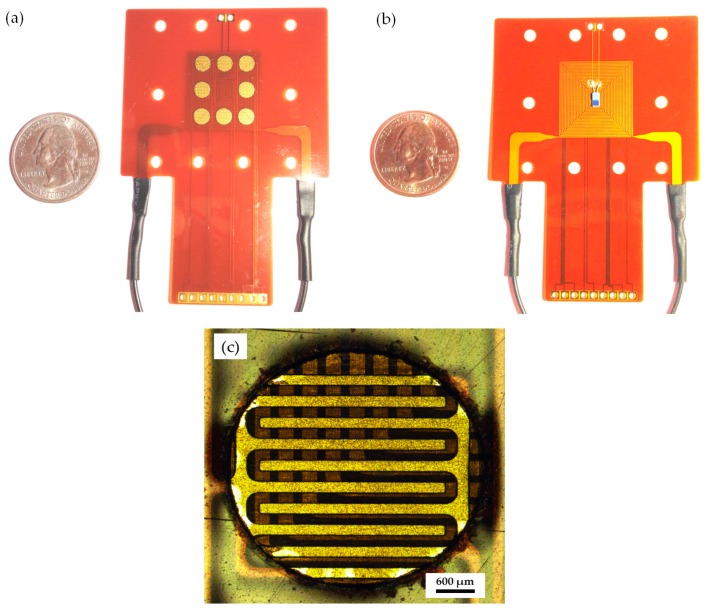
Configuration of flexible gas sensor array: (**a**) Front photograph of gas sensor array; (**b**) Reverse side photograph of heater embedded with RTD sensor; (**c**) Photograph of interdigitated electrodes.

Ambient temperature variation is one of the several variables that pose critical problems in reducing the sensitivity of the sensor. A previous study demonstrated that higher operating temperature reduces sensor response [31]. To protect the polymer/MWCNT composite gas sensor array from the influence of temperature, it was equipped with a heater composed of stainless steel (SUS304, thickness 50 μm) to provide a thermostat operating temperature. The heater was designed in a double-spiral shape with an area of 20 × 20 mm^2^. A suitable operating temperature was set and monitored by using a platinum RTD. The interdigitated electrode was composed of copper measuring 35 μm in thickness and having a finger gap size of 200 μm. The configuration of the flexible gas sensor array is shown in Figure 3.

### 3.2. Gas-Sensing System Architecture

**Figure 4:**
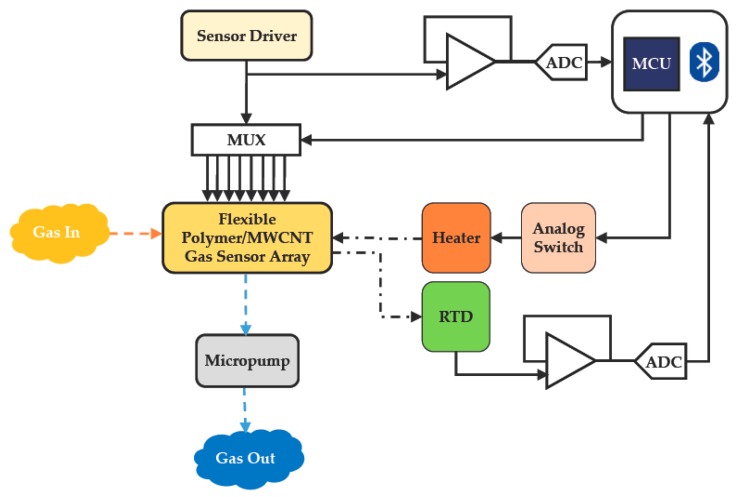
System block diagram of wearable and wireless gas-sensing system.

Figure 4 shows a functional block diagram of the proposed wireless and wearable gas-sensing system. Odor was introduced into the gas chamber when the micropump was driven, and the polymer/MWCNT composite films were operated at the thermostat temperature. The polymer film swelled up slightly when the odor was introduced; then, the odor molecules could penetrate into the polymer through the interface pores on the MWCNT surface. The conductivity of the MWCNTs changed owing to charge transfer between the electron-donating/electron-withdrawing molecules [32,33,34]. This led to a change in the distance between the MWCNTs. The change in the resistance of the sensor was measured using sensor interface circuitry and translated to an analog-to-digital converter with a 32-bit microcontroller. The data was transmitted to a smartphone or tablet via wireless Bluetooth communication. The sensor response of each polymer displayed on the smartphone application (app) was normalized and presented as a bar chart.

**Figure 5:**
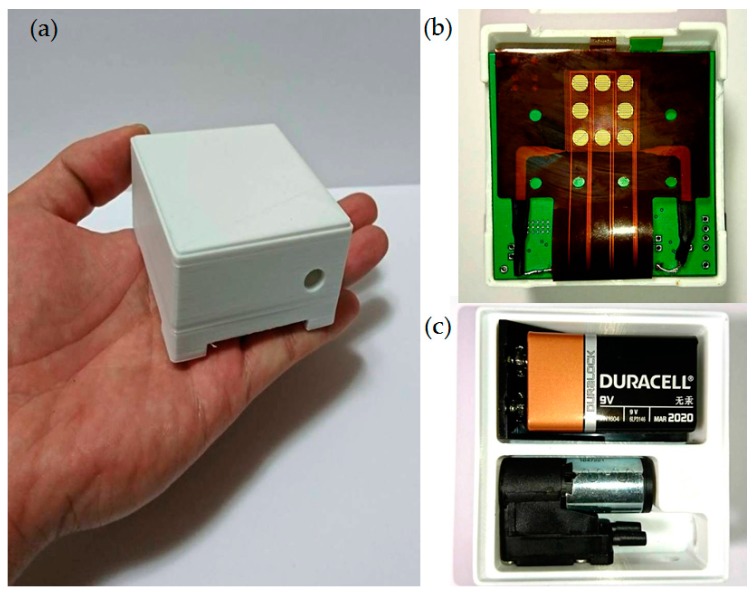
Prototype of wearable and wireless gas-sensing system. (**a**) Photograph of wearable and wireless gas-sensing system; (**b**) Front photograph of gas sensor array; (**c**) Reverse side photograph of micropump and battery.

The system was powered by a single alkaline battery of desired capacity with a nominal voltage of 9 V. Figure 5 shows the proposed wearable and wireless gas-sensing system.

**Figure 6:**
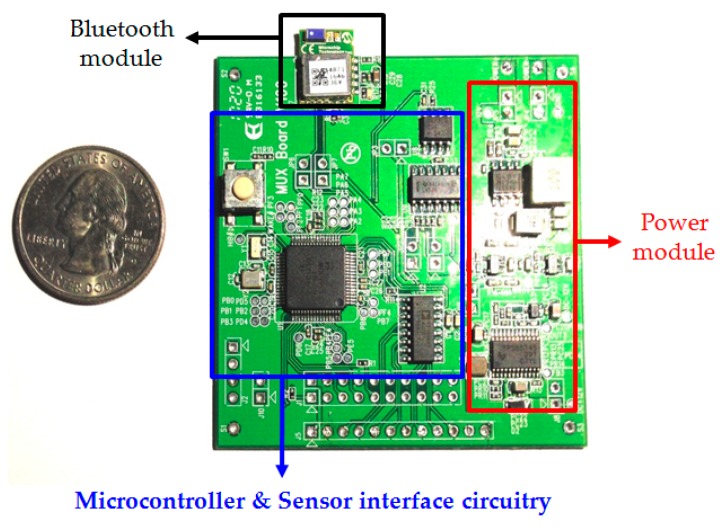
Designed wireless sensor readout module board.

Bluetooth wireless technology is widely used to substitute traditional cable-linked electronic devices [27,35,36,37]. With respect to the proposed system, a Bluetooth module, sensor interface circuitry, and microcontroller (ARM Cortex-M4F, Texas Instruments, Dallas, TX, USA) were integrated into a wireless sensor readout module board for wearable applications, as shown in Figure 6. A Bluetooth^®^ 4.2 Low Energy (BLE) module from Microchip (RN4871-VRM118) was used as the Bluetooth transmitter to acquire and transmit the sensor data to a smartphone.

### 3.3. Smartphone Application

**Figure 7:**
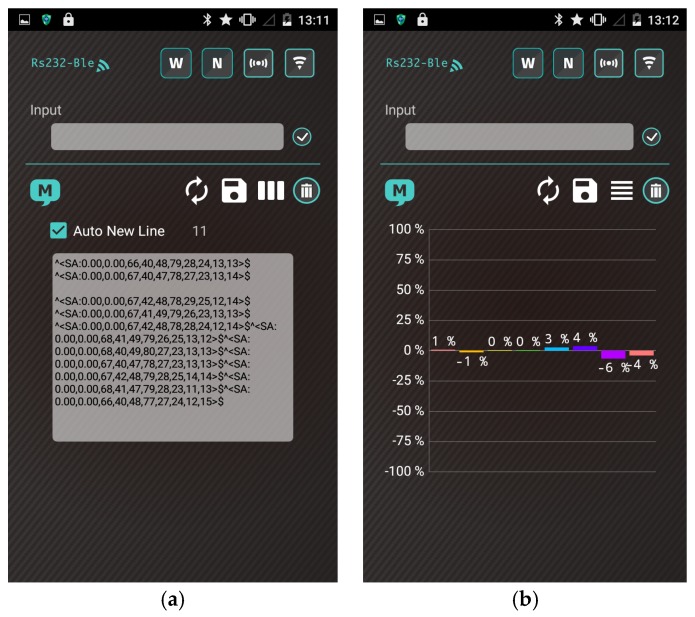
Smartphone application developed for wearable and wireless gas-sensing system. (**a**) The stream of sensor data; (**b**) Bar chart graph.

Figure 7 shows the smartphone app with a user-friendly interface for data display. The app was programmed in the Android environment to accompany the proposed gas-sensing system.

The operation procedures of the smartphone app are as follows:

(1) The user turns on the gas-sensing system, and the application establishes a secure Bluetooth connection with the gas-sensing system.
(2) The heater remains stable at the operating temperature (30 s). Subsequently, the app receives a stream of sensor data under the normal condition in real time from the gas-sensing system, as shown in Figure 7a.
(3) A real-time bar chart graph of the normalized values with respect to the selected polymers for sensing activities is constructed and displayed in the app, as shown in Figure 7b. The sensor response data are refreshed at intervals of 1 s. Table 2 presents the polymer/MWCNT composite sensors and the relative bar chart channel numbers (from left to right).
(4) The app continuously logs sensor response for 10 min. After the subjects complete the task, the app clears the data and resets to step (2) for a new cycle.
(5) These data and graphs are stored on the device and uploaded to cloud servers online.

## 4. Results and Discussion

### 4.1. Heater Performance

**Figure 8:**
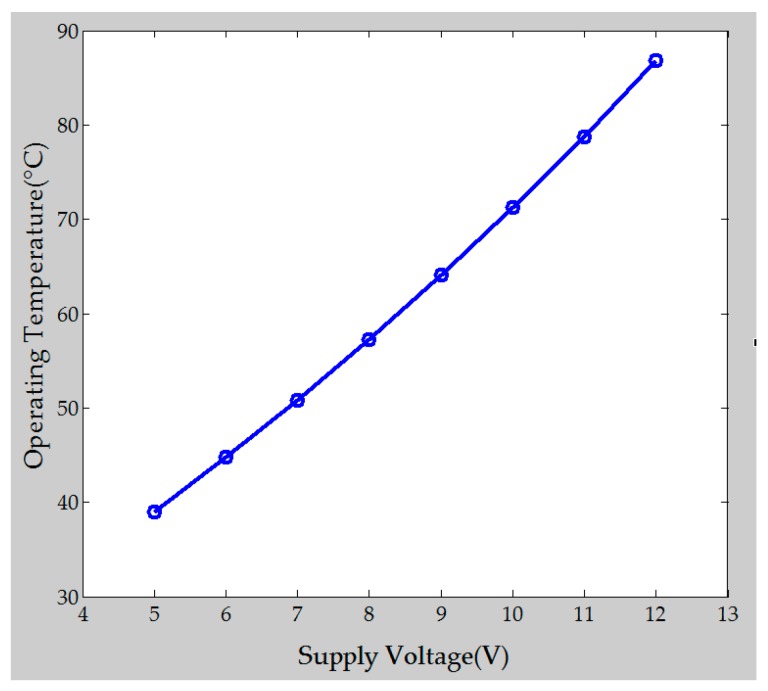
Operating temperature with respect to applied heater voltage.

To protect the polymer/MWCNT composite gas sensor array from the influence of ambient temperature variation, a heater was embedded in the flexible gas sensor array as a thermostat. The operating temperature range of the heater as a function of applied voltage (5–12 V) in the flexible gas sensor array is shown in Figure 8.

**Figure 9:**
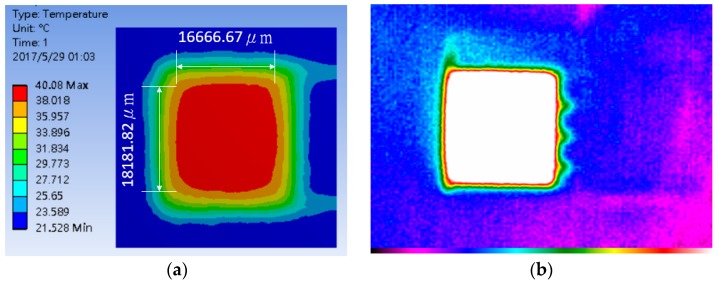
Thermal distribution of flexible polymer/multi-walled carbon nanotube sensor array: (**a**) Simulation result; (**b**) Thermographic measurement.

To validate the heater design, a steady-state electro-thermal simulation was conducted via finite element analysis in ANSYS to realize the thermal distribution. Figure 9a clearly shows the central heating area in the gas sensor array when the temperature is fixed to 40 °C. Temperature uniformity over the heated area size of the heater corresponds approximately to 18,181 μm × 16,666 μm, as shown in Figure 9a. Additionally, the heater was used in a thermal image camera (Thermoteknix Systems Ltd, MIRICLE 307K-25, Cambridge, UK) to measure the thermal distribution. The results of the infrared thermal image are consistent with the results of the ANSYS simulation.

### 4.2. Thermal Stability

**Figure 10:**
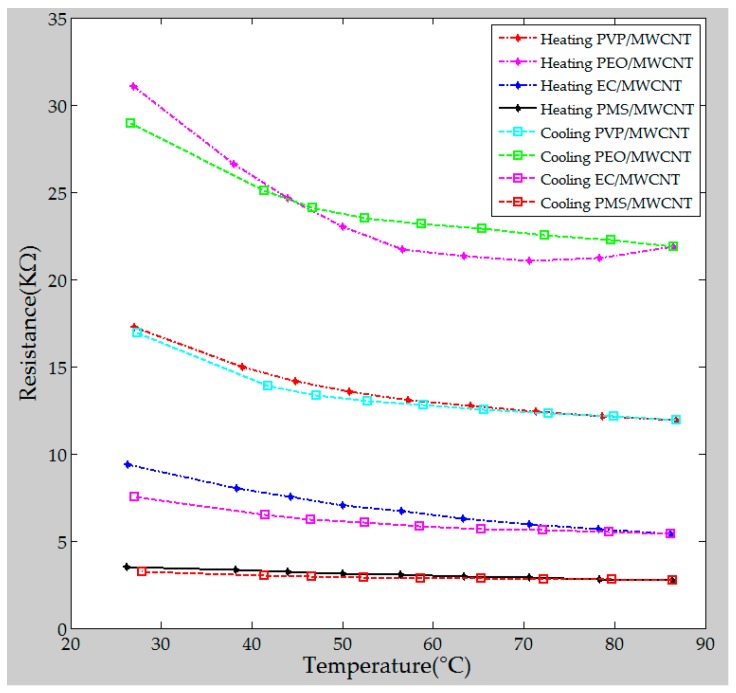
Resistance variation of each polymer/MWCNT composite film with respect to temperature (heating–cooling profile).

An efficient method for protection from the influence of ambient temperature allowed for sustained use of the polymer/MWCNT composite sensing film at a suitable operating temperature. The effect of heating on the resistance of the polymer/MWCNT composite sensing film was examined using a heating–cooling cyclic protocol [38]. Figure 10 shows the heating–cooling profile of the polymer/MWCNT composite sensing film. The temperature of the heating–cooling profile was in the range of 27–86–28 °C. The resistances of the PEO/MWCNT and the EC/MWCNT composite films suffered from drift when compared with those of the remaining two polymers. Therefore, with respect to operation of the gas sensor array, the temperature was set to 40 °C to reduce the influence of ambient temperature. The heater embedded in the sensor array consumed approximately 210 mW at 40 °C.

### 4.3. Sensing Performance

**Figure 11:**
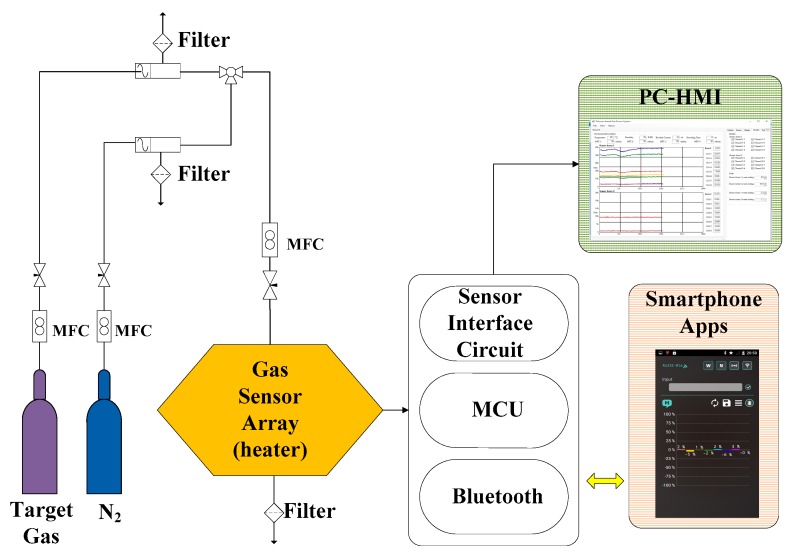
Experimental setup of sensing response system.

The sensor response of the polymer/MWCNT composite gas sensor array was evaluated by measuring the change in its resistance upon exposure to an analyte (target gas) and nitrogen (N_2_, 99.99%, background gas). The flow rates of both the target gas and the background gas were controlled to under 350 mL/min by using a mass flow controller. The experimental setup used in the measurements is shown in Figure 11. Sensor responses were acquired by using the sensor interface circuitry and then transmitted to a smartphone via the Bluetooth module. The smartphone app calculated the collected data and then divided the relative change in the resistance of the polymer/MWCNT composite films by the baseline resistance. Finally, a bar chart of the normalized resistance changes (Δ*R*/*R*%) was displayed in real time on the smartphone.

The gas detection process included several steps. First, nitrogen gas was introduced into the reaction chamber for 10 min to obtain a reference resistance baseline. Simultaneously, the flexible polymer/MWCNT composite gas sensor array was heated to an operating temperature of 40 °C. Subsequently, the target gas was introduced into the reaction chamber for 5 min. The polymer films swelled because they adsorbed the gas molecules [39,40,41,42]. The conductivity of the MWCNTs changed owing to charge transfer between the electron-donating/electron-withdrawing molecules [32,33,34]. After the target gas reacted with the gas sensor array, nitrogen gas was introduced for 10 min to enable desorption from the polymer film. In this work, three target gases were employed, namely, ammonia (NH_3_), nitrogen dioxide (NO_2_), and toluene (C_7_H_8_). We used high concentrations of NH_3_ and NO_2_ to understand the characteristics of the MWCNTs. To further understand the response of the polymer/MWCNT composite gas sensor to VOCs, we used low concentrations of toluene for testing.

**Figure 12:**
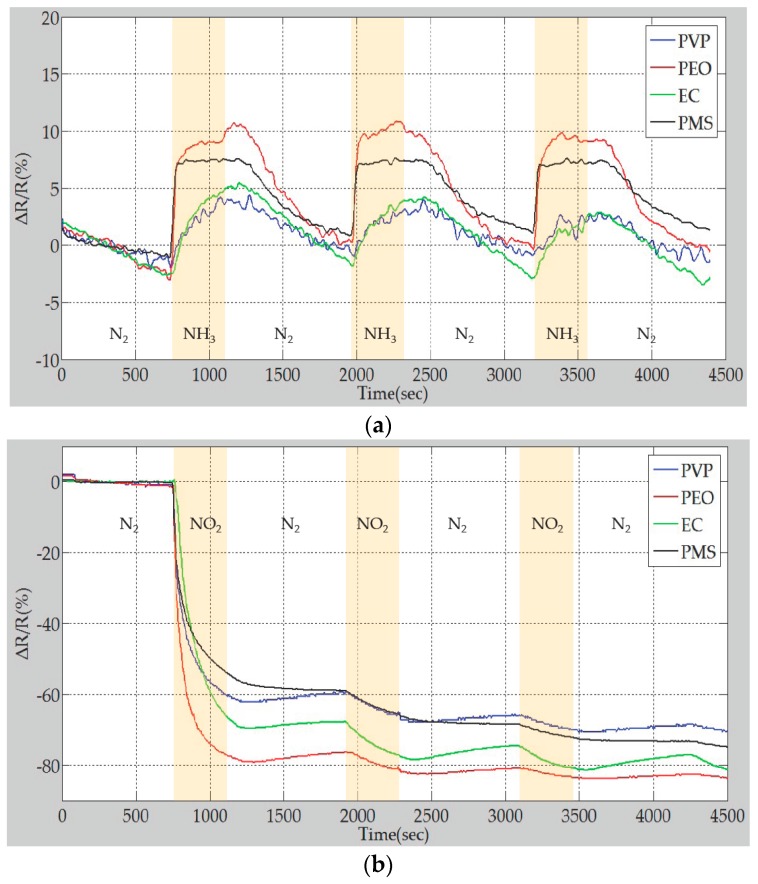
Repeatability test of gas sensor array using different gases. (**a**) Response of the gas sensor array to 1000 ppm NH_3_; (**b**) Response of the gas sensor array to 1000 ppm NO_2_.

Figure 12 shows the repeatability test of the sensor response in the presence of both 1000 ppm NH_3_ and 1000 ppm NO_2_.

Based on the results of the repeatability test, the MWCNTs possess p-type semiconductor characteristics when exposed to two different target gases. The response changed when electron donor (NH_3_) or acceptor (NO_2_) gas molecules were introduced to the polymer/MWCNT composite gas sensor array. NH_3_ gas donates electrons to the surface of the MWCNTs, thus decreasing the number of positive holes in the MWCNTs and increasing the resistivity of the MWCNTs. In contrast to NH_3_, NO_2_ withdraws electrons from the surface of the MWCNTs and decreases their resistivity [33,34]. The different responses of the polymer/MWCNT composite sensing layer showed that the adsorption of gas molecules and the subsequent swelling of the polymer caused the charge transfer to change to varying degrees.

**Figure 13:**
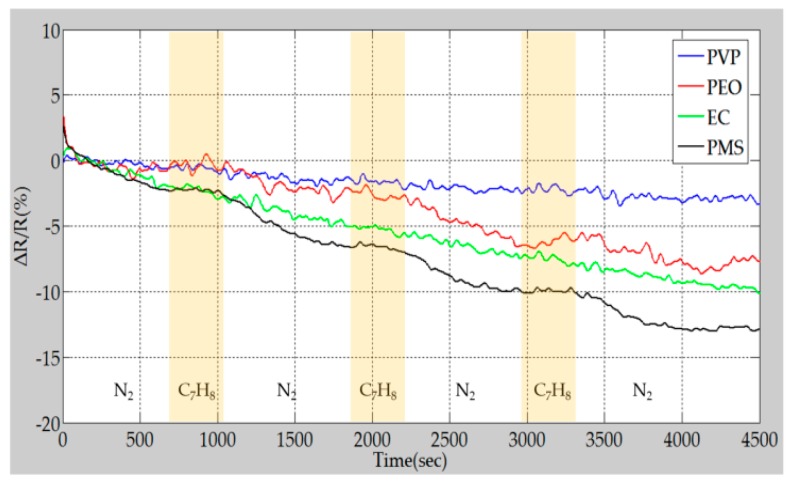
Repeatability test of gas sensor array at 10 ppm toluene.

Figure 13 shows the results of the sensor response repeatability test in the presence of 10 ppm toluene (C_7_H_8_). Although the PVP/MWCNT and EC/MWCNT sensors did not exhibit any response to 10 ppm toluene, the PEO/MWCNT and the PMS/MWCNT sensors exhibited a more obvious response under the same experimental conditions.

The change of resistance of the PEO/MWCNT sensor can be ascribed to two possible mechanisms: one is weak interaction between toluene molecules and MWCNTs and the other is swelling of the PEO [40]. In the case of the PMS/MWCNT sensors, it may be because the functional group of toluene is similar to that of PMS, so toluene is easily adsorbed by PMS, which induces a higher response than that of other polymer/MWCNT sensors. Based on to the response of the polymer/MWCNT composite gas sensor array to toluene gas, the sensor array was used to construct a toluene vapor pattern to ensure good selectivity.

### 4.4. Smartphone App Communication

**Figure 14:**
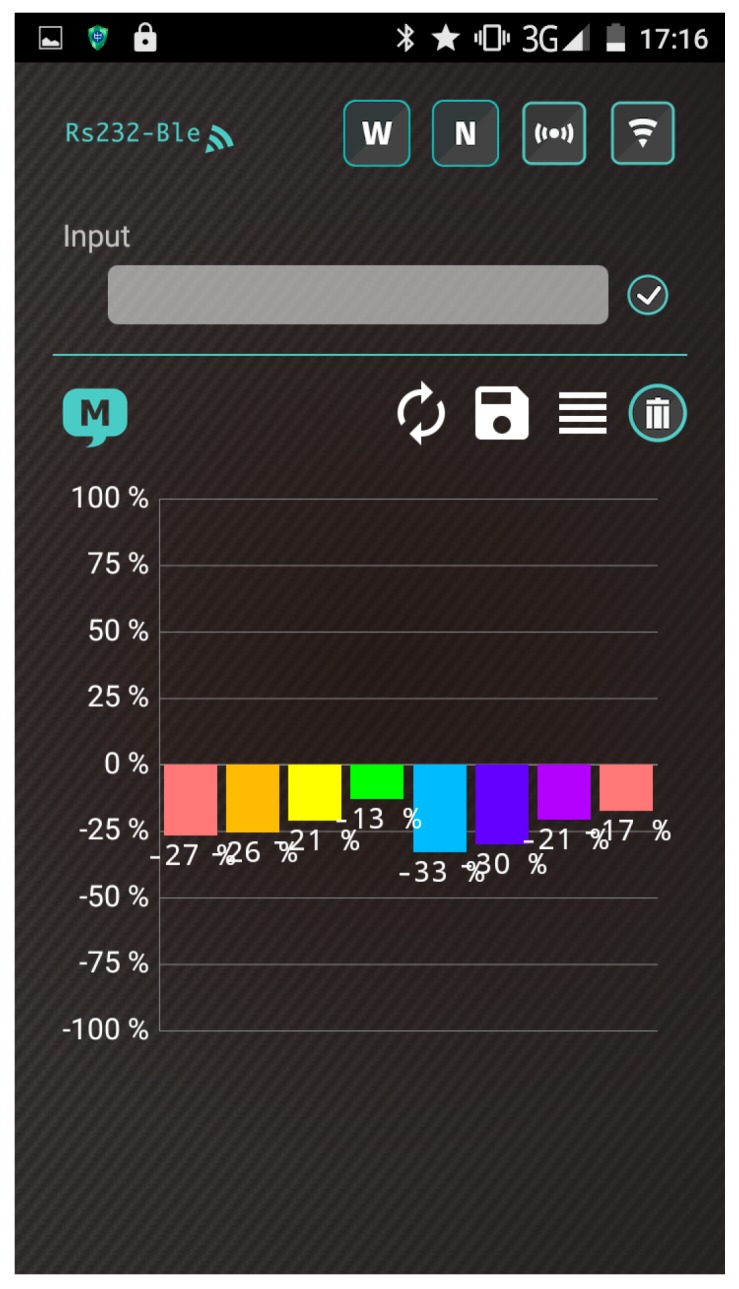
Response of gas sensor array when heated to operating temperature.

A wearable and wireless gas-sensing system based on a Bluetooth wireless module design indicates the feasibility of recognizing the behavior of a polymer/MWCNT composite sensor array. Figure 14 shows screenshots obtained from the proposed system apps after heating the array to the operating temperature. When the system was heated to the operating temperature, all sensors in the system exhibited strong negative responses.

**Figure 15:**
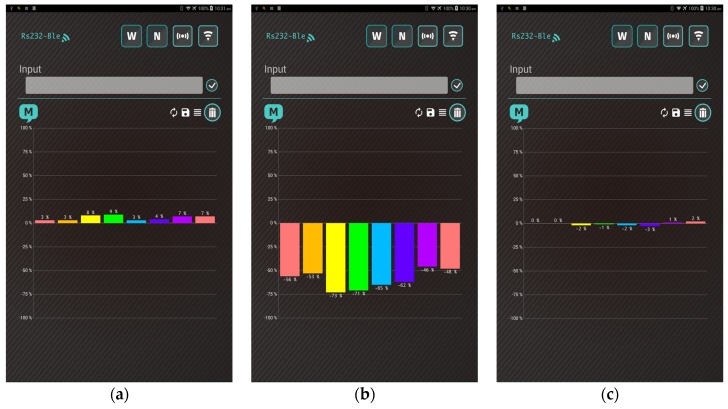
Screenshot of sensor response pattern to different operating conditions: (**a**) exposure to 1000 ppm ammonia; (**b**) exposure to 1000 ppm nitrogen dioxide; (**c**) exposure to 10 ppm toluene.

Figure 15 shows the response patterns of the polymer/MWCNT composite gas sensor array under different conditions, including ammonia, nitrogen dioxide, and toluene. When the system was exposed to electron donor (NH_3_) and acceptor (NO_2_) gaseous molecules, the response patterns were opposite owing to the p-type semiconductor characteristics of the MWCNTs. When the system was exposed to toluene, the PEO/MWCNT and PMS/MWCNT arrays sensed a response, while PMS/MWCNT arrays revealed slightly response owing to baseline drift.

## 5. Conclusions

In this study, we fabricated a flexible polymer/MWCNT composite sensor array based on FPC technologies and the drop-casting method. A heater was embedded in the flexible gas sensor array to serve as a thermostat to protect it from the influence of ambient temperature. Four different polymers were used to manufacture eight sensors for sensing ammonia, nitrogen dioxide, and toluene vapors. Bluetooth technology was used to achieve real-time sensing and to display the sensor response on a smartphone. A future study will involve principal component analysis for responsive pattern recognition. The proposed system is a promising candidate for deployment in a wearable gas-sensing system used to study air pollution.

## Figures and Tables

**Table 1:** Details of polymers in sensor array.

Sensor Name	Sensing Film	Element Number
PVP1	1 wt % PVP/MWCNT	3,1
PVP2	1 wt % PVP/MWCNT	2,1
PEO1	1 wt % PEO/MWCNT	1,1
PEO2	1 wt % PEO/MWCNT	3,2
EC1	1 wt % EC/MWCNT	1,2
EC2	1 wt % EC/MWCNT	3,3
PMS1	1 wt % PMS/MWCNT	2,3
PMS2	1 wt % PMS/MWCNT	1,3
*	Resistance Temperature Detector (RTD)	2,2

**Table 2:** Details of polymer/MWCNT composite sensors in smartphone app.

**Channel No.**	1	2	3	4	5	6	7	8
**Sensor Name**	PVP1	PVP2	PEO1	PEO2	EC1	EC2	PMS1	PMS2
